# Ecotoxicology in the Context of Biodiversity Loss: Lessons from Seven Decades of Chemical Impacts and Paths Forward

**DOI:** 10.1021/acs.est.6c00982

**Published:** 2026-05-29

**Authors:** John P. Sumpter, Luigi Margiotta-Casaluci

**Affiliations:** † Brunel University of London, Uxbridge UB8 3PH, United Kingdom; ‡ Institute of Pharmaceutical Science, Faculty of Life Science and Medicine, 4616 King’s College London, London SE1 9NH, United Kingdom

**Keywords:** ecotoxicology, biodiversity loss, chemical pollution, environmental risk assessment, regulatory science, chemical mixtures, NAMs

## Abstract

Chemical pollution is a major anthropogenic driver of biodiversity loss, yet its relative contribution compared to other stressors remains difficult to quantify. Ecotoxicology emerged as a discipline in response to evidence that chemicals in the environment can harm wildlife, but anticipating and preventing ecological damage remains challenging. This Review examines how ecotoxicology research has informed environmental protection, drawing on case studies spanning over 70 years, including pesticides and birds, tributyltin and mollusks, diclofenac and vultures, and 6PPD-quinone and salmon. These examples highlight recurring challenges—such as unpredicted species-specific sensitivities, unanticipated exposure pathways, and modes of action overlooked by standard testing frameworks—that have typically resulted in reactive rather than preventive regulatory responses. In light of the thousands of chemicals in use and widespread environmental mixtures, the Review evaluates strengths and limitations of current ecotoxicological testing and regulatory practices. It proposes pragmatic principles for enhanced protection, emphasizing prevention, prioritization under uncertainty, improved predictive capacity, and cross-sector collaboration, while acknowledging inevitable trade-offs between environmental safeguards and essential societal uses of chemicals. The Review argues that ecotoxicology must evolve rapidly by embracing predictive non-animal and data-driven approaches to more effectively reduce the risk of severe, widespread, or irreversible ecological harm.

## Graphical abstract

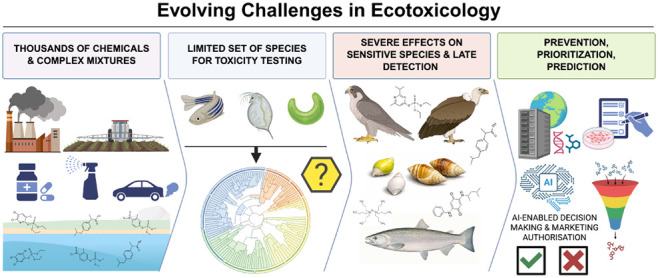

## Introduction

Biodiversity underpins the structure, functioning, and resilience of ecosystems, sustaining fundamental planetary processes as well as the ecosystem services on which human societies depend.^1^ Nevertheless, biodiversity faces a wide variety of significant stressors, including habitat loss and fragmentation, competition from alien (introduced) species, new and emerging diseases, anthropogenic hunting and harvesting, climate change, and pollution. It is extremely difficult to rank these stressors in any consistent or universal way, because their relative importance differs among species, ecosystems, and geographical regions. At a global scale, recent quantitative syntheses indicate that land and sea use change and direct exploitation are the dominant direct drivers of recent biodiversity loss, with pollution playing a more or less dominant role depending on the biodiversity component considered and the ecological context.^2^ Specifically, those analyses revealed that pollution ranks second among five key drivers for effects on genetic composition, ecosystem function and structure, and third for species traits, whereas it appears to play a smaller role (compared to other stressors) in determining community composition and species populations. Nonetheless, the contribution of pollution to biodiversity loss is likely to be underestimated, partly because it is exceptionally difficult to quantify the cumulative pressure exerted by exposure to complex mixtures of (thousands of) chemicals at ecologically relevant spatial and temporal scales.

Some well-documented examples (see “Past Problems” section below) demonstrate that chemical pollution can have, and has had, devastating effects on some species. Historically, ecotoxicology has therefore been defined as the scientific study of the fate and effects of chemical stressors on living organisms, particularly at the population, community, and ecosystem levels, with the aim of understanding and, where possible, anticipating adverse ecological effects. In recent years, however, ecotoxicology has increasingly been framed within a broader societal context in which biodiversity conservation is widely recognized as a primary environmental protection goal.^3^ In this context, advances in ecotoxicology have been explicitly linked to efforts to protect biodiversity and ecosystem services, particularly through improved detection, prediction, and management of chemical risks.^4^ This Review therefore provides a critical assessment of ecotoxicology as a field, drawing on representative examples to identify recurring themes and lessons from past experience, to evaluate how effectively ecotoxicological knowledge has informed regulatory decision-making, and to consider how the discipline might evolve to better address the challenges posed by chemical pollution within the broader context of biodiversity loss.

## Past Problems

The historical examples described below involve cases in which the causes of severe environmental damage were eventually identified and, in some instances, regulatory action was taken. However, these cases also highlight the difficulty of identifying and mitigating serious environmental risks in a timely manner (Figure 1). In each example, chemicals caused profound harm to wildlife before their effects were recognized, leading to outcomes that included population collapses, local extinctions, and major disruption of ecosystems. Importantly, none of these outcomes was anticipated by the ecotoxicological testing or environmental risk assessment frameworks in place at the time. Moreover, it remains uncertain whether such effects would be anticipated even under the most advanced regulatory practices currently in place.

**Figure 1:**
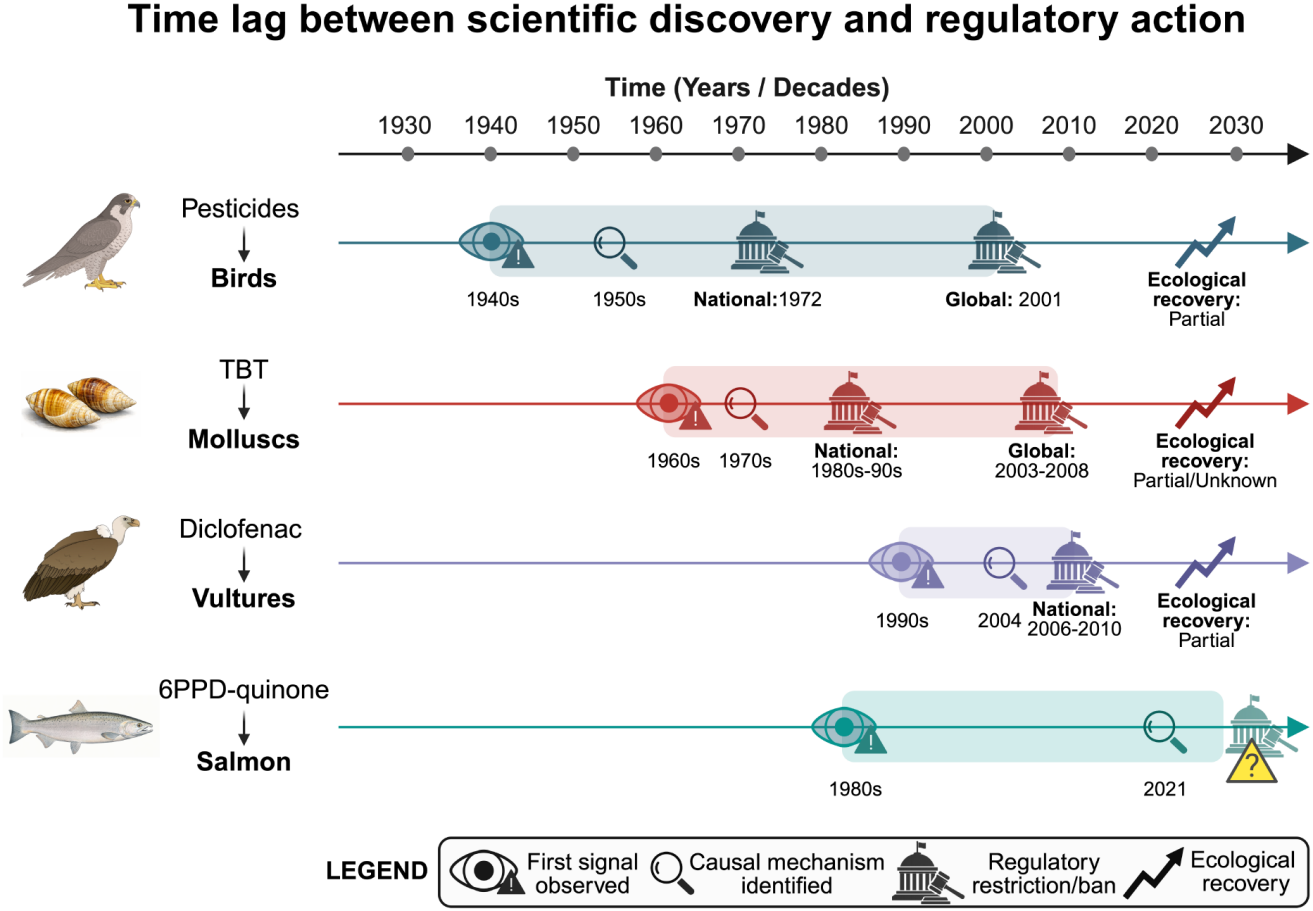
Time lag between ecological observation, cause identification, and regulatory action for selected high-impact chemical pollution case studies. Timelines illustrate the approximate delays between initial observations of adverse ecological effects (“first signal”), identification of the causal chemical agent, and implementation of regulatory restrictions for four well-documented cases: pesticides and birds, tributyltin (TBT) and mollusks, diclofenac and vultures, and 6PPD-quinone and salmon. In all cases, substantial delays—ranging from years to several decades—separate early ecological warnings from cause identification and effective regulatory action. Ecological recovery has been partial, uncertain, or not yet observable, reflecting the persistence of some chemicals and the challenges of reversing widespread environmental contamination. Dates and durations are indicative and based on the published literature.

### Pesticides and Birds

One of the earliest well-documented examples of chemicals present in the environment adversely affecting wildlife was the case of pesticides and predatory birds. In the late 1940s, peregrine falcon populations in several countries began to show signs of precipitous decline. It was demonstrated shortly afterward that it was the widespread use of a range of cyclodiene pesticides, including aldrin, dieldrin, and heptachlor, which were being used as seed dressing in the UK and elsewhere, that were responsible.^5–7^ These pesticides biomagnified up the food chain, to accumulate to lethal concentrations in raptors, including peregrine falcons. At the same time there was also widespread mortality of seed-eating birds in locations where cyclodiene pesticides were heavily used.^8^ In addition, DDT caused eggshell thinning, leading to eggs breaking during incubation.^9^ These severe effects led to the subsequent banning of the pesticides responsible. This case represents the earliest example of the use of chemicals being banned after severe effects on wildlife were recognized following their widespread use and consequent contamination of the environment. The broader social recognition of pesticide harms was catalyzed by Rachel Carson’s book Silent Spring in 1962,^10^ which documented effects of DDT and other organochlorine pesticides on wildlife and helped to stimulate policy attention to environmental toxicity. In the United States, after nearly a decade of scientific and regulatory review following that publication, the U.S. Environmental Protection Agency issued a cancellation of most agricultural uses of DDT in 1972, although exemptions for public health use (e.g., malaria control) were maintained. Subsequently, throughout the late 1970s and 1980s, many cyclodiene pesticides (such as aldrin, dieldrin, heptachlor, and chlordane) were progressively banned or withdrawn in the U.S., the European Union, and other countries. At the global level, most of these chemicals (including DDT and the cyclodienes) were listed under the Stockholm Convention on Persistent Organic Pollutants, adopted in 2001 and entering into force in 2004, effectively banning or restricting their production and use worldwide.^11^

### Tributyltin (TBT) and Mollusks

TBT is an organotin biocide that was commonly and widely used across the world in antifouling paints for boats and larger ships from the 1960s onward. It was very effective at preventing organisms attaching to the underside of boats. However, a serendipitous discovery about a decade later^12^ led ultimately to the realization that TBT caused a condition called imposex in marine gastropod mollusks: females developed male genitalia, leading to sterility. In turn, populations of marine gastropods declined worldwide, even becoming extinct in some places. TBT concentrations as low as 1 ng/L caused imposex traits in some mollusk species. The compound was also shown to cause shell thickening and deformities in oysters at a concentration as low as 2 ng/L.^13^ Despite the early recognition of severe biological effects, regulatory action was slow. The first restrictions on TBT use were introduced in France in 1982,^14^ primarily to protect the oyster industry, more than a decade after adverse effects had been reported. Surveys from the 1980s onward demonstrated that imposex in mollusks—arguably the most sensitive taxonomic group—was widespread internationally, including in locations distant from harbors and major shipping lanes. To date, over 200 mollusk species have been shown to be adversely affected by TBT.^15,16^ Although international support for a ban on TBT emerged in the late 1990s, a global prohibition was only implemented under the International Maritime Organization’s antifouling convention between 2003 and 2008.^17^ Subsequent monitoring has shown that mollusk populations are recovering in some areas, although many TBT “hotspots”, such as harbors and docks, remain heavily contaminated due to the compound’s slow degradation rate.^18^ It took many years, and numerous false leads, before the primary mechanism of action of TBT was identified as agonism of the retinoid X receptor (RXR),^19^ implicating endocrine disruption via a pathway highly conserved across the animal kingdom. Consequently, TBT has been shown to adversely affect a wide range of aquatic taxa, including crustaceans, ascidians, and fish. The full ecological consequences of releasing TBT into the marine environment will almost certainly never be known. Reflecting this legacy, TBT has been described by some ecotoxicologists as one of the most hazardous anthropogenic chemicals ever intentionally released into the environment.^18^

### Diclofenac and Vultures

A precipitous decline in the populations of a number of species of vulture in Southeast Asia in the 1990s was eventually linked to the veterinary use of the nonsteroidal anti-inflammatory drug (NSAID) diclofenac.^20,21^ Diclofenac was being used to treat pain and fever in cattle. When treated cows died, their carcasses were left in the open and consumed by vultures. It was observed that Asian vultures in the genus Gyps are exquisitely sensitive to diclofenac, which causes rapid kidney failure and death within a few days. By early 2002, vulture populations had declined by over 95%, with some species experiencing losses up to 99%, making these probably the fastest and most dramatic population crashes of any species of bird.^22^ Millions of vultures died before the cause was identified and acted upon. Importantly, this was not a consequence of environmental persistence as diclofenac degrades relatively rapidly in the environment. Rather, the catastrophic effects resulted from continuous exposure through a highly specific and previously unrecognized pathway, combined with extreme species sensitivity and species-specific pharmacokinetic profile (i.e., longer drug half-life compared to humans).^23^ Subsequent research demonstrated that other NSAIDs, including ketoprofen and nimesulide, are also highly toxic to Gyps vultures. In contrast, some alternative NSAIDs, such as meloxicam, are not,^24,25^ likely reflecting differences in pharmacodynamics and metabolic handling. To add further complexity, New World vultures (e.g., the North American turkey vulture) appear to be relatively insensitive to diclofenac, highlighting pronounced interspecific differences in avian sensitivity to NSAIDs.^24^

Regulatory action followed relatively rapidly after the causal link between diclofenac and vultures mortality was established;^20,21^ however, ecotoxicology research on this matter intensified only after the scale of the population collapse became severe.^26^ Veterinary use of diclofenac was banned in India in 2006, with Nepal and Pakistan implementing similar bans later that year, and Bangladesh following in 2010.^27^ By this point, vulture populations had already been reduced to a tiny fraction of their former size. Conservation programmes, including captive breeding and reintroduction, are now underway, but recovery is slow due to the vultures’ low reproductive rates and the continued illegal use of toxic NSAIDs in some regions. In addition to the direct ecological impacts, the loss of vultures had significant indirect consequences. Feral dog populations increased due to greater food availability, contributing to a resurgence of rabies and associated human mortality.^28^ This case illustrates how the loss of a single functional group can propagate through ecosystems, with profound consequences for both ecological integrity and human health.

### 6PPD-Quinone and Salmon

This is the most recent of the four cases and remains unresolved. For several decades, it had been reported that there were mass die-offs of coho salmon spawning in urban streams in the Pacific Northwest of the US.^29^ It was suggested that the culprit was washing off nearby roads during heavy rainfall, but it took an impressive piece of ecotoxicological research to identify the culprit as 6PPD-quinone, a transformation product of the antioxidant and antiozonant 6PPD used in tires to prevent their degradation, formed through reaction with ozone.^30^ As tires wear, they shed particles that contain many different chemicals, including 6PPD-quinone, which is readily mobilized during storm events.

It transpired that coho salmon (*Oncorhynchus kisutch*) are exquisitely sensitive to 6PPD-quinone, with mortality occurring rapidly at low ng/L concentrations. Other salmonid fish are somewhat less sensitive, including other *Oncorhynchus* species such as chum salmon and cutthroat trout, while small fish species commonly used in regulatory ecotoxicology, such as zebrafish (*Danio rerio*) and fathead minnow (*Pimephales promelas*), are less sensitive by two or more orders of magnitude. Invertebrates appear to be less sensitive still (reviewed in Gonzalez-Vazquez et al., 2025).^31^ In this respect, the case closely parallels that of diclofenac and vultures: a highly visible and ecologically important species exhibits extreme sensitivity to a chemical that appears comparatively benign when assessed using standard test species. As neither coho salmon nor vultures are routinely included in regulatory toxicity testing, such effects would have been very difficult to anticipate.

As in previous examples, moving from ecological observation to causal identification required several decades and regulatory responses remain incomplete. As of now, the use of 6PPD is not banned in either the United States or the European Union. Nevertheless, the discovery has prompted substantial regulatory scrutiny, litigation, and the development of mitigation strategies aimed at reducing environmental release, particularly via stormwater pathways. Whether these measures will be sufficient to prevent further population-level impacts on sensitive salmon species remains uncertain.

Taken together, these examples, spanning more than seven decades, illustrate that the most serious environmental impacts of chemicals emerged from a combination of factors that are difficult to capture within standard ecotoxicological frameworks: extreme sensitivity of particular species or taxonomic groups, exposure pathways that were not considered relevant at the time, and modes of action that fell outside the scope of routine toxicity endpoints. In several cases, the species most severely affected were not those typically used in laboratory testing. They also illustrate that the interval between early scientific signals, causal attribution, and effective regulatory action has ranged from several years to multiple decades, with intervention typically occurring only after severe impacts had already become apparent (Figure 1). These examples also demonstrate that regulatory action, when it occurred, was largely reactive, following clear evidence of environmental harm, rather than preventative. Even in the most recent cases—where modern tools enabled relatively rapid identification of a causal agent—regulatory responses have remained incomplete and exposure has continued. These lessons are not confined to the past, but they highlight enduring challenges for ecotoxicology.

## The Scale of the Issue

Worldwide, there are thought to be around 350,000 registered chemicals.^32^ Although it is unclear how many of these are in regular use, it is likely to be a significant proportion. Once a chemical is in use, it is probably inevitable that it will eventually contaminate the environment. Some chemicals will degrade quite quickly, although they may be constantly entering the environment, but others are very persistent; some are even considered to be “forever chemicals”, due to their extreme resistance to degradation (e.g., per- and polyfluoroalkyl substances, PFAS).

The number of different chemicals reported to date to be present in the environment appears to be unknown, but it is probably in the thousands.^33^ Recent advances in analytical chemistry now enable pg/L concentrations of chemicals to be reliably identified and measured, and the increasing use of nontarget screening approaches continues to reveal large numbers of previously unrecognized contaminants. In addition to the “parent” chemicals, the environment will also be contaminated with their transformation products formed through biotic and abiotic processes. There are likely to be many more transformation products in the environment than there are “parent” chemicals (see, for example, Henning et al., 2021).^34^ In some cases, these transformation products can be as persistent, mobile, or biologically active as the parent compound, and in other instances more so. For example, whereas the nonionic nonylphenol polyethoxylated surfactants are not estrogenic, their major environmental degradation product nonylphenol is both estrogenic and toxic.^35^ It is therefore highly probable that the chemical complexity of the environment is far greater than is usually acknowledged.

This chemical diversity must be considered alongside the extraordinary biological diversity potentially exposed. Between 5 and 11 million species of living organisms (or even more) are estimated to live on our planet, including 88,000+ mollusks, 83,000+ crustaceans, 37,000+ fish and 11,000+ birds.^36^ Even within relatively well-studied taxa, such as vertebrates, there is enormous diversity in physiology, life history, and ecological role. It is clearly impossible to test the effects of more than a minute fraction of environmental chemicals on more than a very small number of species. Consequently, ecotoxicology relies on extrapolation: from a limited number of chemicals to many others, and from a limited number of test species to the vast majority of organisms in the environment.

Exposure further complicates this already complex picture. Environmental contamination is essentially universal: from densely populated urban regions to the Arctic and Antarctic, ecosystems are now exposed to complex and ill-defined mixtures of chemicals that were not present until very recently in evolutionary terms (most for less than one century). Further complications arise from questions about how many of those chemicals enter organisms, the extent to which they are taken up, and whether they bioconcentrate and biomagnify through food webs. These toxicokinetic processes depend on their physicochemical properties and on the biology of the organisms exposed, determining which chemicals are likely to reach internal concentrations capable of causing biological effects. However, our ability to characterize these processes across large numbers of chemicals and species remains limited.

Finally, when considering chemical contamination of the environment, it is important to note that not all chemicals contribute equally to ecological risk. Hence, one of the most difficult questions an ecotoxicologist needs to answer, before beginning any research, is which chemical, or group of chemicals, to focus on. While much of the recent and current ecotoxicology research is being conducted on organic chemicals (as exemplified by the four examples discussed above), recent evidence suggests that some metals may be of greater importance in affecting macroinvertebrate diversity and abundance in rivers than many organic contaminants.^37^

Taken together, these considerations highlight the scale and complexity of the challenge facing ecotoxicology. The number of chemicals in use, the diversity of organisms potentially exposed, and the ubiquity of complex mixtures make it impossible to rely solely on traditional, chemical-by-chemical and species-by-species testing approaches. Addressing this reality requires accepting that prioritization is unavoidable and that ecotoxicology must increasingly focus on identifying those chemicals, exposure scenarios, and biological targets most likely to result in significant ecological harm. How this can be achieved, and what lessons can be drawn from past experience regarding the predictive capacity of ecotoxicology, are considered in the following section.

### Key Lessons Learned

It could be argued that because relatively few chemicals that reach the environment have been conclusively demonstrated to cause adverse effects on wildlife, then the vast majority of those 350,000 chemicals appear to be benign from an environmental perspective. In our opinion, this is an untenable conclusion. The historical examples discussed above suggest that environmental harm caused by chemicals is usually detected only when effects are severe, widespread, and occur in species that are visible, high profile, charismatic or of particular ecological or societal importance: dead vultures or dead salmon do not go unnoticed, especially when mass deaths occur. But the vast majority of species in the world do not receive much, if any, attention. Distributions and population sizes of most species are poorly documented; in fact, the majority of living species have not even been named. Complete loss of such species could easily go unnoticed; certainly specific, localized, populations could decrease dramatically, or disappear completely, without anyone ever noticing. Consequently, the apparent rarity of documented impacts is more likely to reflect limitations in detection and monitoring than true absence of effect. Only comprehensive monitoring of ecosystems and the many species within them could alleviate this issue, and that is very unlikely to occur, even in the rich, developed, countries.

A second, closely related, lesson is that species-specific sensitivity fundamentally limits the predictive power of standard ecotoxicological testing. The higher sensitivity of Gyps vultures to diclofenac mentioned in the previous sections, compared to other vultures and (nonvulture) bird species, provides a striking example of how very similar species can demonstrate very different sensitivities to the same chemical. These cases demonstrate that reliance on a small number of surrogate species, although unavoidable in practice, will inevitably fail to identify highly sensitive taxa. This is not a criticism of the use of standard test species per se, but rather a recognition of its structural limitations. On the other hand, it also highlights the importance of advancing scalable computational models to predict species sensitivity that could be deployed to a number of species much larger than the one currently employed in experimental laboratory testing (i.e., see LaLone et al., 2021 and Margiotta-Casaluci et al., 2024 for, respectively, the presentation of recent international initiatives to advance cross-species extrapolation, and a critical discussion of the state-of-the-art of this field of research).^38,39^ Environmental risk assessments (ERAs), even when conducted to meet all regulatory requirements, are based on responses to very few organisms: often one species of fish, one invertebrate, and one plant. Such ERAs are likely to determine the general toxicity of a chemical, which may read across to many other species, but they are not designed to identify adverse effects occurring in particularly sensitive species or taxonomic groups that fall outside the testing framework. The high sensitivity of Gyps vultures to diclofenac and some other NSAIDs provides an excellent example of this dilemma.

We have learned that uptake of chemicals into organisms, and their propensity to bioconcentrate, can be very important factors in toxicity. A chemical is unlikely to cause effects if it cannot enter an organism, and uptake is largely governed by physicochemical properties such as hydrophobicity.^40,41^ Hydrophobic chemicals therefore often warrant particular attention, although chemicals that are not strongly hydrophobic, such as diclofenac (its LogD value at neutral pH is reported to be 1.31) can still lead to major adverse environmental effects. We have also learned that once a chemical has been internalized by an organism, if that organism is then eaten by a predator, it can pass up the food chain, a process termed biomagnification. This can lead to the highest concentrations of a chemical being present in top predators. For example, contamination of killer whales (orcas), the apex predator of the marine environment, with polychlorinated biphenyls (PCBs) can lead to individual animals containing tens, or even hundreds, of grams of PCBs.^42^ Such high body burdens of PCBs (and other persistent chemicals) probably explain why many populations of killer whales are reproducing poorly, if at all.^43^

The four examples cited above of chemicals causing adverse effects on the environment are all examples of single chemicals causing effects. But there is a distinct possibility that mixtures of chemicals could cause adverse effects, even when each of the chemicals present in the mixture would not, in itself, cause the effect. It has been shown conclusively, in a laboratory experiment, that a mixture of chemicals can inhibit reproduction of fish when each chemical was present at a concentration that, by itself, would not have inhibited reproduction; a phenomenon termed “something for nothing”.^44^ Although such an effect would be extremely difficult to demonstrate in the natural environment, modeling studies have led to the suggestion that the complex mixture of organic chemicals present in rivers in Europe is adversely affecting their biodiversity.^45^ It is also likely that the presence of intersexuality in wild fish in many rivers in the UK receiving wastewater effluent^46^ was a consequence of a number of different estrogenic chemicals acting together.

Taken together, these lessons—drawn from chemically diverse compounds, affecting a wide range of terrestrial and aquatic species, and operating at regional to global scales—suggest that ecotoxicology must operate with a clear understanding of its limitations in predicting rare but high-impact ecological effects. Protecting biodiversity from chemical pollution cannot rely solely on standardized testing of individual chemicals in a small number of model species. Instead, it requires approaches that better integrate exposure, toxicokinetics, species sensitivity, mixture effects, and ecosystem functioning, and that are capable of prioritizing those chemicals and scenarios most likely to result in serious ecological damage. How current testing strategies attempt to address these challenges, and where they fall short, is considered next.

## Ecotoxicological Testing of Chemicals

Ecotoxicological testing is conducted primarily to support environmental (or ecological) risk assessment (ERA), which aims to determine the potential of a chemical to adversely affect organisms likely to be exposed in the environment. Central to this process is the derivation of a predicted no-effect concentration (PNEC), defined as the concentration of a chemical that is not expected to induce adverse effects in organisms receiving chronic (including permanent) exposure. To derive PNECs, chemicals are tested for their effects on a limited number of representative taxa, typically including primary producers (most often algal species rather than higher plants), invertebrates (such as crustaceans, insects, or mollusks), and vertebrates (most often fish). Where sufficient chronic toxicity data are available for multiple species, species sensitivity distributions (SSDs) can be constructed to estimate protective concentrations. When data are more limited, assessment factors are applied to account for uncertainty (e.g., interindividual and interspecies variability, laboratory to field extrapolation, etc.), reflecting both scientific limitations and policy judgment.^47^ Presently there is no universal agreement on the size of assessment factors, or which issues they should cover, and hence the process of applying assessment factors, though understandable and defensible, is somewhat arbitrary.

Early ecotoxicological tests focused on acute mortality, but over time it became evident that lethality alone was an inadequate measure of environmental risk, and chronic tests based on endpoints such as growth and reproduction were developed. The rationale underpinning such tests is that many different modes of action (of the test chemicals) will lead to changes in development, growth and/or reproduction, which have very high ecological relevance. Many chronic bioassays have been standardized and validated by the Organization for Economic Co-operation and Development (OECD), which provides comprehensive testing guidelines detailing how such tests should be conducted, and they remain central to regulatory ecotoxicology. Use of standardized protocols should ensure that reliable, and hence repeatable, results are obtained by any ecotoxicologists following the guidelines. Recent advances in omics-related methodologies (e.g., transcriptomics, metabolomics, proteomics) have facilitated and accelerated the inclusion of mechanistic endpoints in ecotoxicity tests, albeit primarily at a research level, rather than in standardized, validated, tests, such as those prescribed by OECD for regulatory purposes.

The reliance on standardized test species and protocols reflects both scientific and practical considerations. Focusing on a few species that are easily maintained allows knowledge to accumulate and results to be compared across studies and laboratories, achieving a degree of reproducibility that is essential for regulatory decision-making.

Besides tests aimed at determining the effects of chemicals (i.e., their toxicity) on organisms, regulatory frameworks require information on the environmental fate of chemicals. Test protocols have been developed to determine the persistence of a chemical in the environment (in water or soil) and their degree of bioconcentration in biota. The rationale behind these tests is that the longer time a chemical persists in the environment (often measured as its half-life), and the more it accumulates in biota, the greater the concern. Those concerns are based on examples such as the PCBs and, more recently, PFAS, the latter often described as “forever chemicals” because they are extremely resistant to degradation once in the environment.

Under most regulatory frameworks worldwide, new chemicals must be assessed for environmental safety before authorization. Regulations differ to some degree from country to country, with the European Union (EU) probably having one of the strictest regulations.

Ethical concerns regarding the use of animals, particularly vertebrates, in toxicity testing have led to increasing pressure to reduce reliance on animal testing.^48–50^ Presently *in vivo* tests utilizing fish embryos and invertebrates (e.g., *Daphnia* species) remain acceptable and are in widespread use. However, the growing societal, political, and scientific pressure has driven substantial investment in the development of alternative approaches, collectively referred to as new approach methodologies (NAMs). Considerable effort is being directed toward developing *in vitro* tests using cell lines, but these undoubtedly have many limitations. Although some are now approved and supported by the OECD (i.e., OECD Test No. 249: Fish Cell Line Acute Toxicity),^51^ their applications to accurately predict chronic (or longer-term) effects remains uncertain and is currently a research focus of an increasing number of research laboratories. A wide range of methodological and technical innovations are aiding the development of new methods to assess both the potential toxicity and bioconcentration of chemicals (these are comprehensively discussed in Langan et al., 2024).^52^ These range from three-dimensional (3D) *in vitro* cell cultures, including spheroids and “organ on a chip”, through predictive in silico methods and models (e.g., physiologically based toxicokinetic modeling (PBTK)), to the use of machine learning and artificial intelligence (AI) algorithms to predict toxicity. Many of these methods provide valuable mechanistic insight and can be applied at a scale that is not feasible with traditional animal testing. In addition, a growing volume of research is trying to understand if ERA based on data generated using nonprotected aquatic invertebrate species could also be protective of fish and amphibians too, limiting the need to perform aquatic vertebrate testing for all chemicals.^53,54^ At present, however, most NAMs for environmental species are not intended (or not ready) to replace standard regulatory ecotoxicity tests. Rather, their greatest potential presently lies in supporting prioritization, read-across, and weight-of-evidence approaches. By helping to identify chemicals with particular modes of action, those likely to be taken up or accumulate in biota, or those that warrant closer scrutiny, NAMs can guide the more targeted use of *in vivo* testing (only when needed) and regulatory resources. Regulatory reluctance to rely solely on ecoNAM-derived data reflects understandable concerns regarding validation, reproducibility, and applicability across diverse chemical classes and biological contexts. It will require broad consensus among scientists, regulators, and other stakeholders before NAMs are readily accepted as able to provide accurate, reproducible information that can be used in the ERA process. Until that is achieved, global regulatory systems will probably remain reliant on data obtained from *in vivo* toxicity tests, including those utilizing vertebrates, especially fish.

Given the scale of chemical contamination and the diversity of organisms potentially affected, it is increasingly clear that ecotoxicological testing cannot proceed on a chemical-by-chemical and species-by-species basis alone. A more integrated and tiered approach would be required, in which mechanistic and computational tools support prioritization and interpretation, exposure and toxicokinetic predictions and data inform relevance, and NAMs and standardized tests provide baseline hazard information. Such approaches do not eliminate uncertainty, but they offer a pragmatic means of managing it.

These realities underscore the challenge at the heart of modern ecotoxicology: the need to inform biodiversity protection using methods that are scientifically defensible, ethically acceptable, and practically feasible, while operating under severe constraints of time, resources, and knowledge. How well current regulatory systems are equipped to address this challenge, and where they fall short, is discussed in the following section.

## Problematic Issues with the Existing Regulatory System

The problems with the current regulatory system for assessing the environmental safety of chemicals have been recognized for many decades. The extremely insightful, and prescient, words of Richard Schoettger, published 45 years ago,^55^ summarized many of the problems that existed then, and still exist today (see Box [Boxed-text box1]). Despite his laboratory having conducted 1,587 acute toxicity tests on 271 chemicals against 28 species of fish and 30 species of invertebrate (a monumental effort that probably could not be replicated by a single laboratory, anywhere, today), he realized that there were nowhere near enough experimental facilities, and appropriately trained ecotoxicologists, available to conduct the testing required at that time. Despite substantial scientific and technological advances since then, this fundamental imbalance between the scale of chemical production and the capacity to assess environmental risk remains largely unresolved.

1 **Quote from “** ***Handbook of** **acute toxicity** **of** **chemicals** **to** **fish** **and aquatic invertebrates*** **”, written in 1980 by Richard A. Schoettger, then Director of the Columbia National Fisheries Research Laboratory**. *“Unfortunately, that is the way things are in the real world, because acute toxicity measurements may be the only aquatic effects data available for many chemicals, and then for only a fraction of the thousands of chemicals that have been identified as having potential to escape into the environment. Ideally, evaluators of potential chemical hazards to the environment would prefer a plethora of additional measurements concerning possible effects on growth, reproduction, pathology, biochemistry, populations of aquatic organisms, and ecological relationships. Frankly, the US scientific community does not have the time, research facilities, trained personnel, experimental animals, nor financial resources to provide the additional data needed for ‘comfortable’ predictions of the possible environmental effects of a broad spectrum of chemical contaminants. What is needed is a strategy for concentrating limited scientific resources on those chemicals most likely to have adverse impacts on aquatic systems.”*

If anything, the situation is significantly worse now. It was reported fairly recently^56^ that aquatic toxicity data are available for only 11% of chemicals registered for use in the European Union, with the situation thought to be similar in the United States. Similarly, Gunnarsson et al. (2019)^57^ estimated that a full set of regulatory compliant data is available for only 12% of pharmaceuticals, which is one of the best studied classes of chemicals. Even if the present situation is somewhat better than those numbers suggest, it still means that tens, or even hundreds, of thousands of chemicals without any environmental information are likely already present in the environment. One reason for this persistent gap is the sheer pace at which new chemicals are introduced. It has been estimated that over 2,000 new chemicals are introduced each year, most of which will eventually reach the environment. Even where regulatory frameworks require preauthorization testing, the resources needed to generate comprehensive environmental data for all chemicals far exceed current global capacity. As a result, regulatory systems are necessarily selective, relying on standardized tests, assessment factors, and conservative assumptions to manage uncertainty. These approaches are defensible and often effective, but they cannot eliminate the risk that important effects will be missed.

To determine the environmental toxicity of a chemical requires three factors to be met: there must be appropriate facilities, adequately trained staff, and money. Most companies manufacturing and/or using chemicals do not conduct their own safety assessments. Many did in the past, but now the testing of chemicals is usually done by contract research laboratories. The global number of such organizations is unknown, but it is likely to be very much lower than the number that would be required if all chemicals were to be adequately tested prior to their use. Unfortunately, a high proportion of the results from this testing of commercial chemicals do not appear in the open, readily accessible, literature, and hence cannot be utilized by other scientists, such as academic researchers.

Academic research plays an important role in advancing ecotoxicology, yet much of it does not translate readily into regulatory decision-making, for a variety of reasons.^58^ Major reasons include the relevance and the quality of the research.^59,60,62^ It is very common to come across ecotoxicology publications in scientific journals that report effects of individual chemicals on single species without sufficient consideration of environmental relevance, adversity, or reproducibility. Very often, the results reported in those studies will be of very little, if any, use to regulators. Some authors have provided explicit guidance on how to conduct and publish the results of ecotoxicology research, in order that they can be used by regulators to protect the environment (e.g., 60, 61, 62, 59). In particular, the Criteria for Reporting and Evaluating Ecotoxicity Data (CRED), published 10 years ago,^61^ have been very influential, and have been recommended for use by several regulatory agencies.^63^ Common problems include published papers not containing crucial information, and/or suffering from serious quality issues. Very few ecotoxicology papers being published meet the “Sound Ecotoxicology” principles outlined by Harris et al. (2014),^58^ even over a decade after those principles, aimed at improving the quality of ecotoxicology research, were published.

Regulatory caution regarding the adoption of new data types and methodologies is frequently cited as a barrier to progress. However, this caution is largely a consequence of regulatory responsibility. Decisions must be legally defensible, transparent, and robust to challenge, particularly when they have significant economic or societal implications. Novel methods that lack standardization, clear applicability domains, or well-understood uncertainties are therefore difficult to incorporate into regulatory frameworks, even when they offer scientific advantages. This conservatism is not unique to ecotoxicology, but it does slow the translation of innovation into practice. Taken together, these factors highlight that the challenges facing regulatory ecotoxicology are not primarily scientific in nature, but structural and institutional. They arise from the need to balance environmental protection with feasibility, consistency, and legal certainty in a context where the number of chemicals far exceeds the capacity for comprehensive assessment. Recognizing these constraints is essential for identifying realistic improvements to the system. The following section considers what practical steps might be taken to strengthen environmental protection within these limits.

## The Way Forward

It is very easy to identify the many problems associated with protecting the environment from chemicals, but extremely difficult to identify a workable, practical way forward^56,64^ that will significantly improve the present situation. It is necessary to accept that synthetic organic chemicals will continue to be manufactured and used, and that some degree of environmental contamination is therefore inevitable. Being realistic, it is unlikely that a perfect regulatory system that would prevent all toxic chemicals (toxic to anything living) reaching the environment can be devised. In that context, the aim of ecotoxicology and regulation cannot be to eliminate all risk, but rather to reduce the likelihood of severe, widespread, or irreversible harm. Table 1 summarizes the major challenges and maps them to five guiding principles for making ecotoxicology more predictive, scalable, and effective.

**Table 1:** Key Challenges for Advancing Ecotoxicology toward Predictive Environmental Protection, Mapped to Proposed Guiding Principles

Guiding Principle	Core Challenge	Why It Matters	Emerging Responses
1. Prevention over reaction	Persistent chemicals become widely distributed before risks are recognized	Once released, mitigation is impractical for decades or longer	Persistence-based screening; class-based restrictions; international convention listings
	Delays of years to decades between scientific evidence and regulatory action	Prolongs exposure and increases likelihood of irreversible harm	Streamlined regulatory triggers; early warning monitoring
2. Reducing environmental release	Continuous input via wastewater, agricultural runoff, and product wear	Maintains chronic ecosystem exposure to complex and poorly characterized mixtures	Advanced wastewater treatment; improved application practices; stormwater management
3. Prioritization under uncertainty	Toxicity data available for only a small fraction of chemicals in commerce	Comprehensive testing is infeasible; decisions must be made with incomplete data	NAMs; AI/ML-based toxicity prediction; large public toxicity data sets; structure–activity approaches
	Mixture effects poorly characterized	Environmental exposure is to mixtures, not single chemicals; cumulative risk is largely unquantified	Mixture toxicity modeling; component-based approaches
	Risk assessment relies on very few model species	Sensitive taxa may fall outside testing frameworks entirely	Cross-species extrapolation models; phylogenetic approaches to predicting sensitivity; biological read-across
4. Quality over quantity	Poorly designed or reported studies consume resources and can mislead	Unreliable findings may take years to correct and erode trust in the discipline	Standardized reporting criteria; improved training in experimental design and statistics
	Testing data often inaccessible or fragmented	Limits reproducibility, independent verification, and cumulative knowledge	Open-access databases; calls for mandatory data sharing
5. Integration and collaboration	Disciplinary and sectoral silos	Slows adoption of new methods; research may not address regulatory needs	Multistakeholder platforms; international testing guideline development
	Fragmented global chemical governance	Chemical pollution is transboundary but regulation is largely national or regional	International science-policy panels; multilateral environmental agreements

These challenges are addressed, in broad terms, by five guiding principles that structure the discussion below. The first and most fundamental is prevention rather than reaction. Once highly persistent chemicals become widely distributed in the environment, there is little that can be done to mitigate their effects for decades or longer. Restricting or prohibiting the manufacture and use of substances that are extremely resistant to degradation could therefore be one of the most effective ways of reducing long-term environmental harm. Although such restrictions are difficult to implement and often controversial, the experience with chemicals such as PCBs, TBT, and PFAS demonstrates the consequences of inaction.

A second principle is reducing environmental release, particularly to aquatic systems, and preventing chemicals reaching the environment in the first place. Wastewater treatment plants are major conduits by which a wide range of chemicals enter rivers, lakes, and coastal waters. Potential actions include improving the efficiency of wastewater treatment plants, reducing unnecessary chemical use, and improving the collection and disposal of waste (which would, for example, help address the global plastic pollution problem). Similar benefits can be achieved through better training and support for users of chemicals, such as farmers, to ensure that products (e.g., pesticides) are applied appropriately and sparingly. Public awareness and pressure can play an important role in driving these changes. For example, increasing public pressure in the UK in response to gross contamination of rivers has forced the regulator (a government organization) to impose strict conditions on the private water companies, with severe financial penalties both to the company and senior managers if these companies fail to reduce their contamination of the aquatic environment.

A third principle is prioritization under uncertainty. Given the scale of the problem, it is neither possible nor desirable to attempt comprehensive testing of all chemicals. Instead, effort should be focused on identifying those substances and exposure scenarios most likely to cause significant ecological harm. This requires integrating information on chemical use patterns, environmental fate, toxicokinetics, and biological activity at molecular and cellular level. NAMs, including *in vitro* assays, mechanistic profiling, high-content screenings, computational models, and machine learning approaches, are particularly valuable in this context.^52^ The concept of prioritization is also essential to tackle one of the greatest real-world challenges in ecotoxicology, environmental, and public health protection, which is the hazard posed by chemical mixtures. Environmental exposure to chemicals is very often to highly complex, ill-defined, mixtures of chemicals. More evidence is needed to demonstrate that chemical mixtures can cause adverse effects even when individual constituents are present at concentrations that would not, alone, cause harm. It may be the case that exposure to one chemical amplifies the effect of another—a well-known phenomenon in human health that can occur with some combinations of pharmaceuticals.

Because it will never be possible to determine by traditional (experimental) means the toxicity of all chemicals proven, or likely, to be present in the environment (see above as to why), it is inevitable that recent advances in machine learning and AI will be utilized to provide predictive ecotoxicology. That is already happening and is likely to play an increasingly important role in providing estimates of the likely environmental toxicity of chemicals. A recent example serves to illustrate what is already possible: Liu et al. (2025)^65^ used existing information on the binding affinities of nearly 60,000 chemicals to over 200 G protein-coupled receptors (these are known to be very important in many physiological processes) to develop a model capable of predicting which chemicals present in plastics were likely to be of concern. That study provided a list of plastic chemicals that could be of high risk and hence warrant regulatory attention. Publicly available large toxicity data sets, such as that generated by the Tox21 program in the USA^66^ are major assets for predictive ecotoxicology. Another example is the ability to predict toxicity via the structure of chemicals, a principle established decades ago when toxicologists were studying the role of chemicals in inducing cancer in people. Recently Gustavsson et al. (2024)^67^ showed that an AI-based model could accurately predict the chronic and acute toxicity of chemicals to aquatic organisms based on the structural features of those chemicals. That approach, which is capable of predicting the toxicity of thousands of chemicals, is supported by the recent finding that the food additive nonivamide is estrogenic, and accounts for a significant proportion of the estrogenic activity in the water of a reservoir in China.^68^ Linking chemical structure, together with other features such as concentrations in the environment and toxicokinetics, to toxicity using computational approaches is likely to be very helpful in prioritizing chemicals for experimental investigation.

As stated above, there is considerable pressure, and desire, to cease using vertebrate species in (eco)­toxicology. A recent paper^69^ makes a significant advance in this process by demonstrating that the toxicity of chemicals is broadly conserved phylogenetically. Using five nonmammalian organisms and a human cell line, they reported that for 72 diverse chemicals there was a high degree of correlation in the rank order of toxicities across the model organisms. Put another way, a chemical that was toxic to a fish would probably be similarly toxic to amphibians, flies, flatworms, crustaceans, and probably much else. There were some species-specific toxicities, but similarities predominated. These findings strengthen the basis for the biological read-across hypothesis,^70^ widening it from just pharmaceuticals to many other groups of chemicals.

A fourth principle is quality over quantity in ecotoxicology research. Poorly designed or poorly reported studies do little to protect the environment and can consume substantial time and resources before their limitations are recognized. There is undoubtedly a need to significantly improve the quality of ecotoxicology research and explicit suggestions have been published on how to improve that situation (see, for example, ref 58). Poor, usually unreliable and unrepeatable, research not only misleads, but also requires a great deal of effort, and many years, to be corrected, as the examples of the pharmaceuticals propranolol and metformin amply demonstrate.^71,72^ Public trust in science is threatened by such occurrences. Equally important is better reporting of research, so that publications contain the information needed for others to assess the robustness of the results.^63^ The environment would be better served by less, but higher quality, research on the potential effects of chemicals. Improving training in experimental design, statistics, and risk interpretation, and aligning research more closely with regulatory and environmental relevance, would substantially increase the value of ecotoxicology as a discipline.^73^ This applies not only to academic researchers, but also to regulators and others involved in interpreting ecotoxicological data.

A fifth principle is integration and collaboration across disciplines and sectors. Presently most research is conducted in silos. Environmental chemists, ecotoxicologists, modelers, ecologists, regulators, and industry scientists often work in relative isolation, despite addressing different aspects of the same problem. Closer collaboration can improve understanding of exposure, facilitate access to relevant data, and ensure that research addresses real-world conditions. Experience has shown that such collaboration can lead to more informative and impactful science, provided it is conducted transparently. The entire ethos and composition of the Society of Environmental Toxicology and Chemistry (SETAC) is based on improving these links.

To synthesize these interrelated challenges and their broader implications, we present a conceptual framework linking key limitations in current ecotoxicological practice to their systemic consequences and to the roles of representative organizations involved in chemical risk assessment and governance (Figure 2).

**Figure 2:**
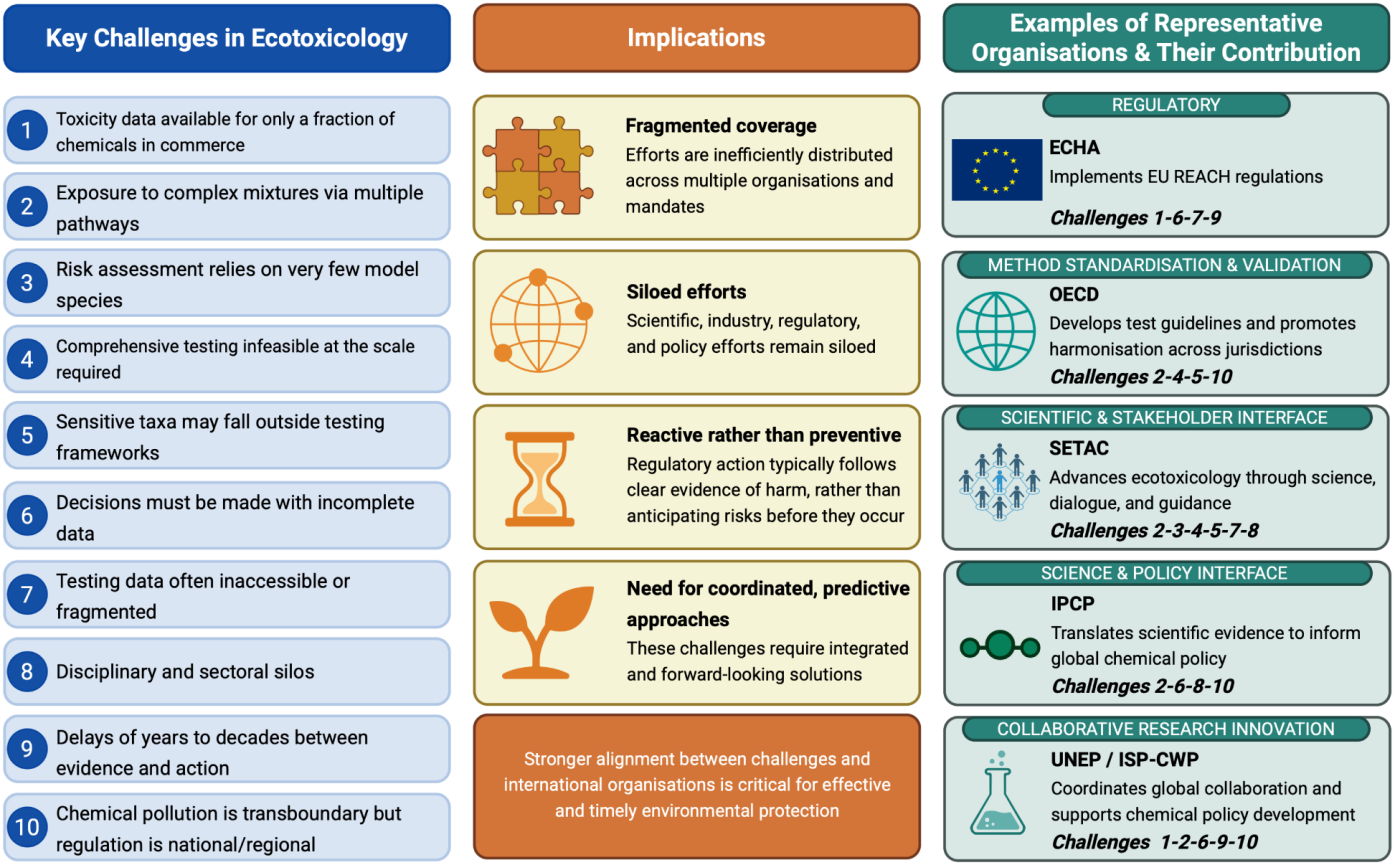
Key challenges in ecotoxicology, their implications, and the roles of representative organizations in addressing them. The figure maps the challenges identified in Table 1 (left) to their broader implications for environmental protection (center) and to examples of organizations contributing to their mitigation (right). The right-hand panel illustrates how different organizations contribute to addressing specific challenges, emphasizing the importance of alignment between scientific, regulatory, and policy frameworks for effective and timely environmental protection. Challenge numbers in the right-hand panel correspond to the numbered challenges on the left. Abbreviations: ECHA, European Chemicals Agency; OECD, Organisation for Economic Co-operation and Development; SETAC, Society of Environmental Toxicology and Chemistry; IPCP, International Panel on Chemical Pollution; UNEP, United Nations Environment Programme; ISP-CWP, International Science-Policy Panel on Chemicals, Waste and Pollution.

Taken together, these principles do not offer a simple solution to the problem of chemical pollution. Rather, they provide a possible pragmatic system-level framework for making incremental but meaningful improvements to environmental protection and enable ecotoxicology to better fulfill its central role in informing decisions aimed at protecting biodiversity and ecosystem health.

## Final Synthesis and Perspective

Ecotoxicology is a relatively new discipline, emerging from the recognition that chemicals released into the environment—both intentionally and unintentionally—can harm wildlife. Although only a limited number of cases have been conclusively documented in which chemical pollution caused severe harm to particular species or ecosystems, it is likely that additional impacts have occurred which have not as yet been detected, and may well never be. Little is known about the population distributions and dynamics of many species, and monitoring is often insufficient. As a result, adverse effects caused by chemical pollution need to be quite severe before they are noticed, meaning that more subtle, yet still ecologically relevant, impacts could easily go unnoticed.

It is difficult to determine the extent to which the ecotoxicology conducted to date has succeeded in preventing environmental problems from occurring, which should be its ultimate objective. In many cases, the discipline has been most effective at identifying problems after they have occurred, rather than anticipating them. This limitation is not unique to ecotoxicology, but reflects the inherent difficulty of predicting biological responses to widespread exposure to large numbers of chemicals under real-world conditions.

These limitations should not detract from the substantial contributions that ecotoxicologists have made to environmental protection. The identification of hazardous chemicals, the elucidation of key mechanisms of action (e.g., endocrine disruption) underlying toxicity, and the development of regulatory frameworks have undoubtedly reduced environmental harm or are likely to do so in the near future (e.g., by facilitating the implementation of derisking strategies within industry chemical discovery programs). A prominent example is the European REACH (Registration, Evaluation, Authorization and Restriction of Chemicals) regulation, which requires companies to register substances manufactured or imported in quantities of one tonne or more per year. Since its implementation in 2007, REACH has resulted in the restriction of 59 categories of chemicals, encompassing more than 1,000 individual substances. However, the examples discussed in this paper demonstrate that reliance on existing approaches alone is unlikely to be sufficient, given the scale of chemical production, the diversity of organisms potentially affected, and the complexity of environmental exposure. In this context, rapid advances in NAMs, coupled with the development of cross-species and AI-enabled predictive models, offer cautious optimism that the ability of ecotoxicology to anticipate and manage ecological risks may improve substantially in the near future—provided these tools are integrated thoughtfully into regulatory and decision-making frameworks.

At the same time, it must be recognized that many of the chemicals of greatest environmental concern underpin essential societal functions, including human and animal health, food production, transport, and infrastructure. Regulatory decisions therefore inevitably involve cost–benefit assessments, often made under considerable ecological uncertainty. Strengthening the ecotoxicological evidence base is thus critical not to eliminate such trade-offs, but to ensure that they are made transparently and with a clearer understanding of long-term, cumulative, and potentially irreversible environmental consequences.

There is nonetheless a strong case for complementing existing regulatory frameworks with broader preventive measures aimed at reducing both the number and the total quantities of chemicals entering the environment, and for more stringent restriction—or outright prohibition—of substances that are extremely resistant to degradation and therefore likely to contaminate ecosystems for decades or longer once released. While regulations such as REACH have substantially improved the identification and control of hazardous substances, they do not fundamentally limit the overall scale of chemical production or guarantee early exclusion of highly persistent chemicals before widespread environmental exposure occurs. Achieving further reductions in long-term environmental risk will therefore require not only regulatory refinement but also sustained public engagement and political commitment. Waiting for clear and unequivocal evidence of environmental harm before acting is unlikely to be a sustainable strategy. Given that increasing numbers of chemicals continue to enter the environment, many of which have been assessed only minimally for their ecological effects, it is very likely that additional problems will emerge. Although it may never be possible to prevent all adverse outcomes, the continued evolution of ecotoxicology—toward greater prioritization, improved prediction, and more proactive risk management—offers the best prospect for reducing the likelihood of severe and irreversible environmental damage in the future.

## References

[1] Oliver T. H., Isaac N. J. B., August T. A., Woodcock B. A., Roy D. B., Bullock J. M. (2015). Declining resilience of ecosystem functions under biodiversity
loss. Nat. Commun..

[2] Jaureguiberry P., Titeux N., Wiemers M., Bowler D. E., Coscieme L., Golden A. S., Guerra C. A., Jacob U., Takahashi Y., Settele J. (2022). The direct drivers of recent global anthropogenic biodiversity
loss. Sci. Adv..

[3] Brooks B. W. (2022). Precision
Ecotoxicology and the Biodiversity Crisis. Environ.
Sci. Technol. Lett..

[4] Fajana H. O., Lamb E. G., Siciliano S. D. (2025). Enhancing
ecological risk assessment
of chemicals for terrestrial ecosystems through ecosystem services
approach. Environ. Toxicol. Chem..

[5] Ratcliffe D. A. (1970). Changes
Attributable to Pesticides in Egg Breakage Frequency and Eggshell
Thickness in Some British Birds. J. Appl.Ecol..

[6] Campbell, L. H. ; Avery, M. I. ; Donald, P. ; Evans, A. D. ; Green, R. E. ; Wilson, J. D. A Review of the Indirect Effects of Pesticides on Birds. JNCC Report No. 227; Joint Nature Conservation Committee: Peterborough, UK, 1997

[7] Greenwood J. (2021). It was not
DDT. British Birds.

[8] Stanley P. I., Bunyan P. J. (1979). Hazards to wintering geese and other
wildlife from
the use of dieldrin, chlorfenvinphos and carbophenothion as wheat
seed treatments. Proc Biol Sci..

[9] Grier J. W. (1982). Ban of
DDT and Subsequent Recovery of Reproduction in Bald Eagles. Science.

[10] Carson, R. Silent Spring; Houghton Mifflin: Boston, 1962.

[11] Stockholm Convention on persistent organic pollutants (POPs); 2018. https://www.pops.int/Portals/0/download.aspx?e=UNEP-POPS-COP-CONVTEXT-2025.English.pdf. (Accessed 13 May 2026).

[12] Blaber S.
J. M. (1970). The
occurrence of a penis-like outgrowth behind the right tentacle in
spent females of Nucella lapillus (L.). J. Molluscan
Stud..

[13] Langston W.
J. (2020). Endocrine
disruption and altered sexual development in aquatic organisms: An
invertebrate perspective. J. Mar. Biol. Assoc.
U. K.

[14] Alzieu C. (1991). Environmental
problems caused by TBT in France: Assessment, regulations, prospects. Mar. Environ. Res..

[15] Vos J. G., Dybing E., Greim H. A., Ladefoged O., Lambré C., Tarazona J. V., Brandt I., Vethaak A. D. (2000). Health
effects of endocrine-disrupting chemicals on wildlife, with special
reference to the European situation. Crit. Rev.
Toxicol..

[16] Giusti A., Barsi A., Dugué M., Collinet M., Thomé J., Joaquim-Justo C., Roig B., Lagadic L., Ducrot V. (2013). Reproductive
impacts of tributyltin (TBT) and triphenyltin (TPT) in the hermaphroditic
freshwater gastropod Lymnaea stagnalis. Environ.
Toxicol. Chem..

[17] Champ M. A. (2000). A review
of organotin regulatory strategies, pending actions, related costs
and benefits. Sci. Total Environ..

[18] Beyer J., Song Y., Tollefsen K. E., Berge J. A., Tveiten L., Helland A., Oxnevad S., Schoyen M. (2022). The ecotoxicology of
marine tributyltin (TBT) hotspots: A review. Mar. Environ. Res..

[19] Nishikawa J., Mamiya S., Kanayama T., Nishikawa T., Shiraishi F., Horiguchi T. (2004). Involvement of the retinoid X receptor
in the development of imposex caused by organotins in gastropods. Environ. Sci. Technol..

[20] Oaks J. L., Gilbert M., Virani M. Z., Watson R. T., Meteyer C. U., Rideout B. A., Shivaprasad H. L., Ahmed S., Chaudhry M. J. I., Arshad M. (2004). Diclofenac residues as the cause of vulture
population decline in Pakistan. Nature.

[21] Shultz S., Baral H. S., Charman S., Cunningham A. A., Das D., Ghalsasi G. R., Goudar M. S., Green R. E., Jones A., Nighot P. (2004). Diclofenac poisoning is widespread in declining vulture
populations across the Indian subcontinent. Proc. Biol. Sci..

[22] zenodo State of India’s Birds, 2023: Range, trends, and conservation status. SoIB Partnership; zenodo, (Accessed 13 May 2026).

[23] Rattner B. A., Whitehead M. A., Gasper G., Meteyer C. U., Link W. A., Taggart M. A., Meharg A. A., Pattee O. H., Pain D. J. (2008). Apparent
tolerance of turkey vultures (Cathartes aura) to the non-steroidal
anti-inflammatory drug diclofenac. Environ.
Toxicol. Chem..

[24] Cook, S. E. ; Green, R. E. ; Lieberherr, E. ; Bowden, C. G. ; Chaudhry, M. J. I. ; Alam, A. S. ; Bharathidasan, S. ; Prakash, V. ; Ghoshal, A. ; Margalida, A. ; Current policies in Europe and South Asia do not prevent veterinary use of drugs toxic to vultures. 2024, 5, e12357. 10.17863/CAM.109219.

[25] Margalida A., Green R. E. (2025). Vulture conservation requires NSAID regulation. Science.

[26] Richards, N. L. ; Gilbert, M. ; Taggart, M. ; Naidoo, V. A cautionary tale: Diclofenac and its profound impact on vultures. In Encyclopedia of the Anthropocene; Elsevier, 2017, Vol. 1–5, pp. 247–255. DOI: 10.1016/B978-0-12-809665-9.09990-0.

[27] Governments of Bangladesh, India, Nepal & Pakistan. Regional declaration on the conservation of South Asia’s critically endangered vulture species; Ministry of Environment and Forests, 2012, https://save-vultures.org/wp-content/uploads/2019/06/2012-Regional-Declaration-on-Vulture-Conservation.pdf (Accessed 13 May 2026).

[28] Frank E., Sudarshan A. (2024). The Social
Costs of Keystone Species Collapse: Evidence
from the Decline of Vultures in India. Am. Econ.
Rev.

[29] Scholz N. L., Myers M. S., McCarthy S. G., Labenia J. S., McIntyre J. K., Ylitalo G. M., Rhodes L. D., Laetz C. A., Stehr C. M., French B. L., McMillan B., Wilson D., Reed L., Lynch K. D., Damm S., Davis J. W., Collier T. K. (2011). Recurrent
Die-Offs of Adult Coho Salmon Returning to Spawn in Puget Sound Lowland
Urban Streams. PLoS One.

[30] Tian Z., Zhao H., Peter K. T., Gonzalez M., Wetzel J., Wu C., Hu X., Prat J., Mudrock E., Hettinger R. (2021). A ubiquitous tire rubber–derived chemical induces acute mortality
in coho salmon. Science.

[31] Gonzalez-Vazquez M. A., Wong B. B. M., Niven R. K., Wlodkowic D. (2025). Organic pollutants
leaching from tire waste: Ecotoxicity implications for aquatic species. Aquat. Toxicol..

[32] Wang Z., Walker G. W., Muir D. C. G., Nagatani-Yoshida K. (2020). Toward a Global
Understanding of Chemical Pollution: A First Comprehensive Analysis
of National and Regional Chemical Inventories. Environ. Sci. Technol..

[33] Muir D. C. G., Getzinger G. J., McBride M., Ferguson P. L. (2023). How Many Chemicals
in Commerce Have Been Analyzed in Environmental Media? A 50 Year Bibliometric
Analysis. Environ. Sci. Technol..

[34] Henning N., Wick A., Ternes T. A. (2021). Biotransformation
of pregabalin in
surface water matrices and the occurrence of transformation products
in the aquatic environment – comparison to the structurally
related gabapentin. Water Res..

[35] Routledge E. J., Sumpter J. P. (1996). Estrogenic activity
of surfactants and some of their
degradation products assessed using a recombinant yeast screen. Environ. Toxicol. Chem..

[36] Ritchie, H. (2022) - “How many species are there?” Published online at OurWorldinData.org.https://archive.ourworldindata.org/20260106-083806/how-many-species-are-there.html. (Accessed 13 May 2026).

[37] Johnson A.
C., Sadykova D., Qu Y., Keller V. D. J., Bachiller-Jareno N., Jurgens M. D., Eastman M., Edwards F., Rizzo C., Scarlett P. M., Sumpter J. P. (2025). Zinc and
copper have the greatest
relative importance for river macroinvertebrate richness at a national
scale. Environ. Sci. Technol..

[38] LaLone C. A., Basu N., Browne P., Edwards S. W., Embry M., Sewell F., Hodges G. (2021). International
consortium to advance
cross-species extrapolation of the effects of chemicals in regulatory
toxicology. Environ. Toxicol. Chem..

[39] Margiotta-Casaluci L., Owen S. F., Winter M. J. (2024). Cross-Species
Extrapolation of Biological
Data to Guide the Environmental Safety Assessment of Pharmaceuticals—The
State of the Art and Future Priorities. Environ.
Toxicol. Chem..

[40] Margiotta-Casaluci L., Owen S. F., Cumming R. I., Polo A. D., Winter M. J., Panter G. H., Rand-Weaver M., Sumpter J. P. (2014). Quantitative Cross-Species
Extrapolation between Humans and Fish: The Case of the Anti-Depressant
Fluoxetine. PLoS One.

[41] Huerta B., Margiotta-Casaluci L., Rodríguez-Mozaz S., Scholze M., Winter M. J., Barceló D., Sumpter J. P. (2016). Anti-anxiety drugs
and fish behavior: Establishing the link between internal concentrations
of oxazepam and behavioral effects. Environ.
Toxicol. Chem..

[42] Remili A., Dietz R., Sonne C., Samarra F. I. P., Letcher R. J., Rikardsen A. H., Ferguson S. H., Watt C. A., Matthews C. J. D., Kiszka J. J., Rosing-Asvid A., McKinney M. A. (2023). Varying diet composition
causes striking differences in legacy and emerging contaminant concentrations
in killer whales across the North Atlantic. Environ. Sci. Technol..

[43] Desforges J., Hall A., McConnell B., Rosing-Asvid A., Barber J. L., Brownlow A., De Guise S., Eulaers I., Jepson P. D., Letcher R. J., Levin M., Ross P. S., Samarra F., Víkingson G., Sonne C., Dietz R. (2018). Predicting
global killer whale population collapse from PCB pollution. Science.

[44] Thrupp T. J., Runnalls T. J., Scholze M., Kugathas S., Kortenkamp A., Sumpter J. P. (2018). The consequences of exposure to mixtures of chemicals:
Something from ‘nothing’ and ‘a lot from a little’
when fish are exposed to steroid hormones. Sci.
Total Environ..

[45] Malaj E., von der Ohe P. C., Grote M., Kuhner R., Mondy C. P., Usseglio-Polatera P., Brack W., Schafer R. B. (2014). Organic chemicals
jeopardize the health of freshwater ecosystems on the continental
scale. Proc. Natl. Acad. Sci. U. S. A..

[46] Jobling S., Nolan M., Tyler C. R., Brighty G., Sumpter J. P. (1998). Widespread
sexual disruption in wild fish. Environ. Sci.
Technol..

[47] Martin O.
V., Martin S., Kortenkamp A. (2013). Dispelling urban myths about default
uncertainty factors in chemical risk assessment – sufficient
protection against mixture effects?. Environ.
Health.

[48] Cronin M. T. D., Berggren E., Camorani S., Desaintes C., Fabbri M., Fabrega J., Herzler M., Ingram J. D. E., Lacasse K., Louhimies S., Maxwell G., Schutte K., Sobanski T., Streck G., Terron A., Worth A. P. (2025). Report
of the European Commission workshop on “The roadmap towards
phasing out animal testing for chemical safety assessments”,
Brussels, 11–12 December 2023. Regul.
Toxicol. Pharmacol..

[49] UK DEFRA (Department for Environment, Food & Rural Affair) (2024) Recommendations for the Adoption of New Approach Methodologies (NAMs) in UK Chemical Regulation; Department for Environment, Food & Rural Affair, 2024, https://assets.publishing.service.gov.uk/media/672cc07deee595f5288bdbea/HSAC_Brief_on_NAMs_-_FINAL_20_Dec2023_PUBLICATION_VERSION.pdf. (Accessed 13 May 2026).

[50] US FDA (Food and Drug Administration) Roadmap to Reducing Animal Testing in Preclinical Safety Studies; 2025, https://www.fda.gov/media/186092/download?attachment (Accessed 13 MAy 2026).

[51] Revel M., Groh K., Bertoli J., Degeratu M.-O., Fischer M., Fischer S., Hoekman J., Jozef B., Li R., Mosimann S. L., Vom Berg C., Županič A., Truffer B., Schirmer K. (2025). Development
of the fish invitrome
for animal-free environmental risk assessment of chemicals. Environ. Toxicol. Chem..

[52] Langan L. M., Paparella M., Burden N., Constantine L., Margiotta-Casaluci L., Miller T. H., Moe S. J., Owen S. F., Schaffert A., Sikanen T. (2024). Big questions to developing solutions:
A decade of progress in the development of aquatic new approach methodologies
from 2012 to 2022. Environ. Toxicol. Chem..

[53] Coors A., Brown A. R., Maynard S. K., Nimrod Perkins A., Owen S., Tyler C. R. (2023). Minimizing Experimental
Testing on
Fish for Legacy Pharmaceuticals. Environ. Sci.
Technol..

[54] Schür C., Paparella M., Faßbender C., Stoddart G., Baity Jesi M., Schirmer K. (2025). Daphnids can safeguard the use of alternative bioassays
to the acute fish toxicity test: A focus on neurotoxicity. Environ. Toxicol. Chem..

[55] Johnson, W.W. ; Finley, M.T. Handbook of acute toxicity of chemicals to fish and aquatic invertebrates: Summaries of toxicity tests conducted at Columbia National Fisheries Research Laboratory, 1965-78; US Department of the Interior, Fish and Wildlife service: Washington, D.C, 1980.

[56] Johnson A. C., Jin X., Nakada N., Sumpter J. P. (2020). Learning from the past and considering
the future of chemicals in the environment. Science.

[57] Gunnarsson L., Snape J. R., Verbruggen B., Owen S. F., Kristiansson E., Margiotta-Casaluci L., Österlund T., Hutchinson K., Leverett D., Marks B., Tyler C. R. (2019). Pharmacology beyond
the patient – The environmental risks of human drugs. Environ. Int..

[58] Harris C. A., Scott A. P., Johnson A. C., Panter G. H., Sheahan D., Roberts M., Sumpter J. P. (2014). Principles of Sound
Ecotoxicology. Environ. Sci. Technol..

[59] Ruden C., Adams J., Agerstrand M. (2017). Assessing
the relevance of ecotoxicological
studies for regulatory decision making. Integr.
Environ. Assess. Manage.

[60] Hanson M. L., Wolff B. A., Green J. W., Kivi M., Panter G. H., Warne M. S., Agerstrand J., Sumpter M. J. P. (2017). How can we make
ecotoxicology more valuable to environmental protection?. Sci. Total Environ..

[61] Moermond C. T. A., Kase R., Korkaric M., Agerstrand M. (2015). CRED: Criteria
for reporting and evaluating ecotoxicity data. Environ. Toxicol. Chem..

[62] Jones L. B., Burns C. J., Arnold K. E. (2025). Systems-based analysis of the factors
affecting the use of academic research in European chemical assessment
and management. Environ. Int..

[63] Moermond C. T. A., Franco A., Casado-Martinez M. C., Kidd K., Korkaric M., Agerstrand M. (2026). Improving
the reporting of ecotoxicity data: Will SETAC
lead the way?. Integr. Environ. Assess. Manage.

[64] Scheringer M., Schulz R. (2025). The state of the world’s
chemical pollution. Annu. Rev. Environ. Resour..

[65] Liu W., Wang H., Fu Z., Cui Y., Chen J. (2025). McGPCR: A
multimodel learning model with improved applicability domain characterization
for predicting G Protein-coupled receptor affinity of plastic chemicals. Environ. Sci. Technol..

[66] Richard A. M., Huang R. L., Waidyanatha S., Shinn P., Collins B. J., Thillainadarajah I., Grulke C. M., Williams A. J., Lougee R. R., Judson R. S., Houck K. A., Shobair M., Yang C., Rathman J. F., Yasgar A., Fitzpatrick S. C., Simeonov A., Thomas R. S., Crofton K. M., Paules R. S., Bucher J. R., Austin C. P., Kavlock R. J., Tice R. R. (2021). The Tox21
10K compound library: Collaborative chemistry advancing toxicology. Chem. Res. Toxicol..

[67] Gustavsson M., Käll S., Svedberg P., Inda-Diaz J. S., Molander S., Coria J., Backhaus T., Kristiansson E. (2024). Transformers
enable accurate prediction of acute and chronic chemical toxicity
in aquatic organisms. Sci. Adv..

[68] Qin Z., Zhou M., Chen Z., Jahnke A., Schäffer A., Shao Y. (2025). Unexpected Discovery of the Food Additive Nonivamide as a Main Estrogenic
Contributor in the Three Gorges Reservoir. Environ.
Sci. Technol..

[69] Hayot G., Lloyd G. R., Diwan G. D., Keith N., Smoot S. R., Cramer von Clausbruch C. A., Kaufman T. V., Barnard M., Tindall A. J., Glaholt S. P., Massei R., Martinez R., Strahle U., Orsini L., Russell R. B., Tennessen J. M., Scholz S., Shaw J. R., Freedman J. H., Colbourne J. K., Weiss C., Dickmeis T. (2025). Alternative vertebrate and invertebrate
model organisms show similar sensitivity as rodents to a diverse set
of chemicals. Environ. Sci. Technol..

[70] Rand-Weaver M., Margiotta-Casaluci L., Patel A., Panter G. H., Owen S. F., Sumpter J. P. (2013). The read-across
hypothesis and environmental risk assessment
of pharmaceuticals. Environ. Sci. Technol..

[71] Blackwell B. R., Ankley G. T., Biales A. D., Cavallin J. E., Cole A. R., Collette T. W., Ekman D. R., Hofer R. N., Huang W., Jensen K. M. (2022). Effects
of metformin and its metabolite guanylurea
on Fathead Minnow (Pimephales promelas) reproduction. Environ. Toxicol. Chem..

[72] Sumpter J.
P., Runnalls T. J., Donnachie R. L., Owen S. F. (2021). A comprehensive
aquatic risk assessment of the beta-blocker propranolol, based on
the results of over 600 research papers. Sci.
Total Environ..

[73] Harris M. J., Huggett D. B., Staveley J. P., Sumpter J. P. (2016). What training and
skills will the ecotoxicologists of the future require?. Integr. Environ. Assess. Manage.

